# Measuring hospital-wide activity volume for patient safety and infection control: a multi-centre study in Japan

**DOI:** 10.1186/1472-6963-7-140

**Published:** 2007-09-03

**Authors:** Kenshi Hayashida, Yuichi Imanaka, Haruhisa Fukuda

**Affiliations:** 1 Department of Healthcare Economics and Quality Management, School of Public Health, Kyoto University Graduate School of Medicine, Yoshida Konoe-cho, Sakyo-ku, Kyoto 606-8501, Japan

## Abstract

**Background:**

In Japan, as in many other countries, several quality and safety assurance measures have been implemented since the 1990's. This has occurred in spite of cost containment efforts. Although government and hospital decision-makers demand comprehensive analysis of these activities at the hospital-wide level, there have been few studies that actually quantify them. Therefore, the aims of this study were to measure hospital-wide activities for patient safety and infection control through a systematic framework, and to identify the incremental volume of these activities implemented over the last five years.

**Methods:**

Using the conceptual framework of incremental activity corresponding to incremental cost, we defined the scope of patient safety and infection control activities. We then drafted a questionnaire to analyze these realms. After implementing the questionnaire, we conducted several in-person interviews with managers and other staff in charge of patient safety and infection control in seven acute care teaching hospitals in Japan.

**Results:**

At most hospitals, nurses and clerical employees acted as the main figures in patient safety practices. The annual amount of activity ranged from 14,557 to 72,996 person-hours (per 100 beds: 6,240; per 100 staff: 3,323) across participant hospitals. Pharmacists performed more incremental activities than their proportional share. With respect to infection control activities, the annual volume ranged from 3,015 to 12,196 person-hours (per 100 beds: 1,141; per 100 staff: 613). For infection control, medical doctors and nurses tended to perform somewhat more of the duties relative to their share.

**Conclusion:**

We developed a systematic framework to quantify hospital-wide activities for patient safety and infection control. We also assessed the incremental volume of these activities in Japanese hospitals under the reimbursement containment policy. Government and hospital decision makers can benefit from this type of analytic framework and its empirical findings.

## Background

Recently in Japan, as in many other countries, several quality and safety assurance activities have been executed through either voluntary actions on the part of individual hospitals, external policy, or legislative pressures [1]. This remarkable movement has resulted from an increasing awareness and demand for patient safety and quality assurance. It has been proposed that the main drivers of this movement come from two sources. One was the beginning of the third party accreditation of hospitals in 1997 through the Japan Council for Quality Health Care (JCQHC) [2]. The other was a shocking medical accident that occurred in 1999, when an attending surgeon mistook one patient for another.

Despite these important developments, there have been few studies aimed at comprehensively analyzing these activities at the hospital level. A primary reason for this has been the difficulty in defining which activities are part of routine medical care and which are specifically part of patient safety and infection control practices. There are two reasons it is critical for a comprehensive methodology to be developed to frame this issue. One is that it is important for both hospitals and policymakers to be able to use a standardized system to evaluate these activities over time across hospitals. Second, a standard measurement framework is central to any analysis of the potential costs and benefits of patient safety practices. These considerations are especially true in Japan, where hospitals typically underutilize critical patient safety practices.

The aims of this study were to measure hospital-wide activities for patient safety and infection control through a systematic framework, and to identify the incremental volume of these activities implemented over the last five years.

## Methods

### Scope of activities for patient safety and infection control

To define the scope of patient safety and infection control practices, we used the concept of incremental activity corresponding to incremental cost. Namely, the incremental activity was defined as the additional patient safety and infection control provided in a hospital in the current year compared to typical activity levels in 1999 (the base case). In other words, we assessed patient safety and quality assurance activities that have been introduced or strengthened since 1999. This approach was used because major developments in patient safety and quality assurance have taken place in Japan since 1999. Although medical advances have also occurred during that time, basic patient care practices have not significantly changed relative to patient safety and infection control. Interest in patient safety practices increased markedly in 1999 following highly publicized medical accidents. These accidents resulted in an analysis of the topic published by the Ministry of Health, Labor and Welfare [3]. This represented the first major action for improving patient safety undertaken by the Japanese government. On the other hand, the accreditation system of infection control doctors (ICD) was also established in 1999 [4,5].

### Hospitals surveyed

Each participating hospital was a certified teaching hospital with more than 300 beds, played a central regional role and provided emergency and intensive care services. Each hospital made strong efforts for patient safety and quality assurance. They were located throughout the country and the ownerships of them were various, including public sector, healthcare corporations, and company. We obtained informed consent from each participating hospital.

This study was approved by the Institutional Review Board of the Faculty of Medicine at the Graduate School of Medicine of Kyoto University (reference number E-5). This survey was conducted from Aug 2005 to Mar 2006.

### Development of measurement framework and questionnaire items

To develop a framework that measures hospital-wide activity for patient safety and infection control, we reviewed findings from past international studies [6-11] and items of the JCQHC hospital accreditation standards. We also collected activity items found on websites and in the public relations materials of a variety of hospitals. We used investigations, interviews with hospital managers and staff in charge of patient safety and infection control, and panel discussions with experts for this assessment. Table 1 shows the framework we developed to measure hospital-wide activity for patient safety and infection control. Domains common to both patient safety and infection control included staff assignment, meetings and conferences, internal review and walk rounds, internal education and training, external education and training, standard manual development, and others. In addition, they included incident reporting, external audits, maintenance of medical equipment, and management of medications for patient safety, and infection surveillance for infection control.

**Table 1:** Framework to measure incremental activity for patient safety and infection control

Domain		Scope and example
	
1.	Staff assignment	Staff assigned to the division of patient safety or infection control to be in charge of their activities
2.	Meetings and conferences	Meetings and conferences held for patient safety and infection control (e.g. supreme decision-making board committee, medical accident investigation committee, regular staff meetings, infection control committee, etc.)
3.	Internal review and walk rounds	Internal check for patient safety or infection control (e.g. review for adherence to manual, clinical chart review, and clinical conferences, etc.)
4.	Internal education and training	Education and training program set up and run inside hospitals (e.g. orientation for new comers, seminars of patient safety or infection control, etc.)
5.	External education and training	Education and training program set up and run outside hospitals (e.g. seminars conducted by government and professional organization, etc.)
6.	Standard manual development	Standardization of processes and manual preparation and revision aimed for patient safety or infection control (e.g. management code and handbook for patient safety or in – hospital infection control, etc.)
7.	Incident reporting	Data collection/analysis as well as measurements of incident reports, adverse events and near misses to assure patient safety
8.	External audit	Third-party evaluation to continuously improve the safety and quality of care (e.g. the Japan Council for Quality Health Care, the International Organization for Standardization, etc.)
9.	Maintenance of medical equipment	Maintenance to prevent medical accidents (e.g. check and repair of medical equipment, etc.)
10.	Management of medications	Management to prevent medical accidents (e.g. medication history management, drug information service, and dispensing instruction, etc.)
11.	Infection surveillance	Data collection, analysis, and measurements of in-hospital infection (e.g. review of medical charts and bacteriologic examination of surgical site infections and catheter-related infections, etc.)
12.	Other activities	Other activities related to patient safety and infection control and not categorized as above activities (e.g. campaigns, issues of public relations, newspapers related patient safety or infection control, etc)

Based on this framework, we developed questionnaire items. For staff assignment, we calculated the amount of time spent working on each specific activity by profession type. Additionally, we gathered activity contents, numbers classified by profession (medical doctor, nurse, pharmacist, other medical staff, clerical employee, and others), the activity time per year, and the frequency of each activity. This data was used to calculate the annual activity in terms of person-time in 2004. For example, in the case of a supreme decision-making board committee on patient safety, we asked how many people belonged to the committee, which specialties the members belonged to, how many hours were spent for the functioning of the committee, and how many times the committee met during 2004. Each activity studied had been introduced or strengthened since 1999, specifically for the purpose of patient safety and quality assurance.

### Subjects and data collection methods

We conducted a self-administered questionnaire and several in-person interviews with the managers of divisions in charge of patient safety and infection control in seven acute care teaching hospitals. In most cases, because responsibilities for patient safety and for infection control were separate, we administered separate questionnaires to the staff and managers of these divisions. We also asked about a variety of staff duties at key locations such as nursing sections, pharmaceutical sections, and administrative sections to participate in this survey, according to need. In order to partially control for differences in the definitions and scope of activities between hospitals, we sent the list of all the collected activity contents to each hospital and requested each to report whether the hospital offered information of all the executing activities.

## Results

Table 2 shows the number of staff assigned to patient safety and infection control divisions or their equivalents, sorted by profession type. Divisions in charge of patient safety were typically staffed by nurses and clerical employees. There were six (85.7%) hospitals with at least one nurse or clerical full-time equivalent (FTE) in these divisions. In almost all hospitals, these types of specialties were assigned. The members of the division in charge of infection control, and the percentage of total working hours spent on these activities per staff varied significantly between hospitals. For example, the percentage of time spent on activities for infection control ranged from 5% to 80% per medical doctor assigned to such a division. Generally, either medical doctors or nurses were the primary staff assigned to these divisions. Unlike patient safety, other medical staff were also assigned in relatively high proportions.

**Table 2:** Numbers of professionals assigned to the patient safety division and the infection control division

		Medical doctor					Nurse					Pharmacist				
Hospital Code			PS division		IC division			PS division		IC division			PS division		IC division	
										
(Number of beds)		N	n	FTE	n	FTE	N	n	FTE	n	FTE	N	N	FTE	n	FTE
			
A	(390)	110	0	(0)	0	(0)	370	0	(0)	0	(0)	20	0	(0)	0	(0)
B	(1,100)	290	3	(0.5)	0	(0)	960	1	(1.0)	0	(0)	60	1	(0.4)	0	(0)
C	(690)	130	0	(0)	0	(0)	510	1	(0.5)	1	(0.5)	20	0	(0)	0	(0)
D	(510)	120	2	(0.2)	1	(0.6)	450	3	(1.2)	0	(0)	20	1	(0.1)	0	(0)
E	(300)	80	1	(0.1)	2	(0.1)	240	1	(1.0)	1	(0.15)	20	0	(0.0)	0	(0)
F	(880)	240	1	(0.1)	1	(0.8)	570	1	(1.0)	1	(1.0)	50	1	(0.1)	0	(0)
H	(520)	100	0	(0)	1	(0.1)	470	1	(1.0)	2	(0.2)	30	0	(0)	1	(0.1)
		Other co-medical staff					Clerical employee					Other staff				
Hospital Code			PS division		IC division			PS division		IC division			PS division		IC division	
										
(Number of beds)		N	n	FTE	n	FTE	N	n	FTE	n	FTE	N	n	FTE	n	FTE
			
A	(390)	270	0	(0)	0	(0)	100	2	(1.85)	1	(0.05)	30	0	(0)	1	(0.08)
B	(1,100)	290	0	(0)	0	(0)	410	1	(1.0)	0	(0)	160	0	(0)	0	(0)
C	(690)	90	0	(0)	0	(0)	40	2	(0.1–0.15)	1	(0.05)	80	0	(0)	0	(0)
D	(510)	110	2	(0.2)	1	(0.8)	50	2	(2.0)	0	(0)	40	0	(0)	0	(0)
E	(300)	90	1	(0.1)	1	(0.7)	80	0	(0)	0	(0)	50	0	(0)	0	(0)
F	(880)	310	0	(0)	0	(0)	330	1	(1.0)	1	(1.0)	190	0	(0)	0	(0)
H	(520)	160	0	(0)	1	(0.1)	160	1	(0.3–0.4)	0	(0)	180	0	(0)	0	(0)

PS: Patient safety

IC: Infection control

N: Total number of each professional at the hospitals

n: Professionals assigned to the patient safety division and the infection control division

FTE: Full-time equivalent (The number of persons after conversion to full time workers)

Table 3 shows the activity breakdown of patient safety by each hospital, and Table 4 shows the activity breakdown of infection control by each hospital. In these tables, "Model_1" and "Model_2" show the average of the seven hospitals calculated after converting each hospital's value into that per 100 beds and that per 100 staff, respectively. Activity for patient safety ranged from 14,557 to 72,996 person-hours across participant hospitals. The mean volumes per 100 beds and per 100 staff were 6,240 person-hours and 3,323 person-hours, respectively. Whereas management of medication was the most common activity in five of the hospitals, either internal review and walk rounds, or internal education and training was the most common activity in the rest of the hospitals. Although the incremental activity associated with medication management was zero in hospital F, there were patient safety and infection control activities in place. These activities had been in place before 1999, however, and thus did not contribute to the incremental increase.

**Table 3:** Incremental activity volume of each activity item by hospital (Patient safety)

								person-hour/(%)	
Hospital Code	A	B	C	D	E	F	H	Model_1	Model_2
Number of bed	390	1100	690	510	300	880	520	100	-
Number of staff	900	2170	870	790	560	1690	1100	-	100

Meetings and conferences	2,102	5,044	2,078	2,247	712	2,238	9,919	591	307
	(5.5)	(6.9)	(13.5)	(6.0)	(2.8)	(15.4)	(24.4)	(9.4)	(9.3)
Internal review and walk round	108	1,875	1,227	578	16,657	2,728	135	911	494
	(0.3)	(2.6)	(8.0)	(1.6)	(66.1)	(18.7)	(0.3)	(14.6)	(15.0)
Internal education and training	2,375	4,354	1,239	5,326	819	4,090	1,636	469	260
	(6.2)	(6.0)	(8.1)	(14.3)	(3.2)	(28.1)	(4.0)	(7.5)	(7.9)
External education and training	2,272	821	132	128	122	79	344	117	54
	(5.9)	(1.1)	(0.9)	(0.3)	(0.5)	(0.5)	(0.8)	(1.9)	(1.6)
Standard manual development	81	267	558	1,017	1,257	359	100	115	67
	(0.2)	(0.4)	(3.6)	(2.7)	(5.0)	(2.5)	(0.2)	(1.8)	(2.0)
Incident reporting	10,896	8,910	3,683	884	1,400	2,243	2,607	791	397
	(28.5)	(12.2)	(24.0)	(2.4)	(5.6)	(15.4)	(6.4)	(12.6)	(12.0)
External audit	1,677	0	0	184	0	866	125	84	39
	(4.4)	(0.0)	(0.0)	(0.5)	(0.0)	(5.9)	(0.3)	(1.3)	(1.2)
Maintenance of medical equipments	2,092	7,741	2,511	5,963	3,420	0	6,172	729	401
	(5.5)	(10.6)	(16.3)	(16.0)	(13.6)	(0.0)	(15.2)	(11.6)	(12.1)
Management of medications	14,117	41,478	3,944	14,578	415	0	16,608	2,022	1,052
	(37.0)	(56.8)	(25.7)	(39.1)	(1.6)	(0.0)	(40.9)	(32.3)	(31.8)
Other activities	2,481	2,505	0	6,355	386	1,955	3,001	434	236
	(6.5)	(3.4)	(0.0)	(17.1)	(1.5)	(13.4)	(7.4)	(6.9)	(7.1)

Total	38,201	72,996	15,372	37,259	25,186	14,557	40,648	6,262	3,307

The proportion of each activity item is shown in parentheses

'Model_1' shows the average of all the seven hospitals calculated after conversion of each hospital's value into that per 100 beds

'Model_2' shows the average of all the seven hospitals calculated after conversion of each hospital's value into that per 100 staff

**Table 4:** Incremental activity volume of each activity item by hospital (Infection control)

								person-hour/(%)	
Hospital Code	A	B	C	D	E	F	H	Model_1	Model_2
Number of bed	390	1100	690	510	300	880	520	100	-
Number of staff	900	2170	870	790	560	1690	1100	-	100

Meetings and conferences	1,848	1,085	723	1,518	543	476	2,145	232	122
	(29.5)	(19.6)	(17.9)	(23.6)	(18.0)	(3.9)	(24.9)	(20.3)	(19.9)
Internal review and walk round	252	1,817	1,638	424	274	497	576	116	69
	(4.0)	(32.8)	(40.5)	(6.6)	(9.1)	(4.1)	(6.7)	(10.1)	(11.3)
Internal education and training	1,973	1,819	322	998	351	3,494	1,150	235	120
	(31.5)	(32.8)	(8.0)	(15.5)	(11.6)	(28.6)	(13.4)	(20.6)	(19.7)
External education and training	1,101	64	712	284	80	1,697	1,099	125	65
	(17.6)	(1.2)	(17.6)	(4.4)	(2.7)	(13.9)	(12.8)	(11.0)	(10.7)
Standard manual development	440	73	144	255	68	167	200	39	20
	(7.0)	(1.3)	(3.6)	(4.0)	(2.3)	(1.4)	(2.3)	(3.4)	(3.3)
Infection surveillance	203	678	312	772	742	2,271	2,370	182	96
	(3.2)	(12.2)	(7.7)	(12.0)	(24.6)	(18.6)	(27.6)	(15.9)	(15.7)
Other activities	454	8	191	2,175	958	3,594	1,060	215	118
	(7.2)	(0.1)	(4.7)	(33.8)	(31.8)	(29.5)	(12.3)	(18.8)	(19.4)

Total	6,271	5,544	4,042	6,426	3,015	12,196	8,599	1,143	610

The proportion of each activity item is shown in parentheses

'Model_1' shows the average of all the seven hospitals calculated after conversion of each hospital's value into that per 100 beds

'Model_2' shows the average of all the seven hospitals calculated after conversion of each hospital's value into that per 100 staff

With respect to infection control, the activity volume ranged from 3,015 to 12,196 person-hours across participant hospitals. This volume was relatively less than that used for patient safety. The mean volumes per 100 beds and per 100 staff were 1,141 person-hours and 613 person-hours, respectively. The most common activity varied by hospital; in three hospitals it was education and in one hospital it was internal review and walk rounds.

**Figure 1:**
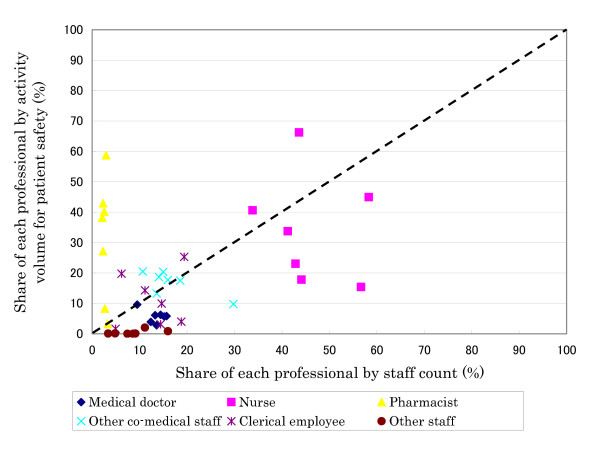
Share of each professional by incremental activity for patient safety and by staff count.

**Figure 2:**
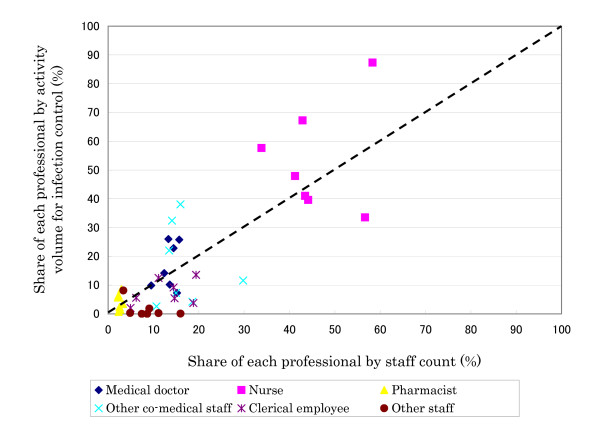
Share of each professional by incremental activity for infection control and by staff count.

Figure 1 and 2 show the share of each professional by staff count against incremental activity for patient safety and infection control, respectively. For patient safety, pharmacists performed more activities than their proportional share in number. On the other hand, for infection control, medical doctors and nurses tended to perform somewhat more of the duties relative to their share. In examining economies of scale, a trend could be found only for nurses, although the sample size was small (detailed data is not shown in this article). This may imply that the more nurses a hospital had, the smaller the volume per nurse.

## Discussion

In this study, we developed a framework for analyzing hospital-wide activity for patient safety and infection control. We systematically measured the volume of activity in seven Japanese hospitals using a developed framework. In the organization of patient safety activities, nurses and clerical employees typically formed the bulk of the staff. In the infection control division, medical doctors and nurses tended to be more often assigned. However, even if the total staff count was calculated by adding all professions' FTE, the ratio of most assigned hospitals remained about one FTE per 315 beds at best. This is lower than other studies, which reported values such as three FTE ICPs per 500 beds (one per 167 beds) [12], one per 178 beds [13], and a recommended standard of one per 250 beds [6]. The volume of activity related to patient safety and to infection control ranged from 14,557 to 72,996 person-hours (per 100 beds: 6,240; per 100 staff: 3,323) and from 3,015 to 12,196 person hours (per 100 beds: 1,141; per 100 staff: 613) across participant hospitals, respectively. These were relatively large volumes considering that the total annual incremental activity per 100 staff was about 4,000 person-hours (i.e. two person-years despite a lack of compensation in reimbursement of medical fees). These results suggest that many investments are needed for quality and safety assurance. Although the most common activity for patient safety was medication management, activities related to infection control varied across the hospitals. In examining the relationship between the share contributed by each type of profession, pharmacists performed a relatively large amount of activities for patient safety in spite of their small numbers. For infection control, medical doctors and nurses tended to perform slightly more activities, though their share of volume was similar to their staff numbers. Previous studies [14,15] have suggested that pharmacists are key players in patient safety, which supports their widespread employment in patient safety activities observed here. In examining the relationship between staff numbers by profession type and activities for patient safety and infection control, the results suggest the availability of economies of scale.

There are several important features of our study. First, we introduced the concept of incremental activity, corresponding to incremental cost, into the extraction of the activity of patient safety and infection control. Using this definition, the scope of activities was specified, and we were able to extract more useful information. This was also made possible by utilizing a unique Japanese context; 1999 was a watershed year in the development of patient safety and infection control practices. This gave a base year for the incremental assessment. Second, we obtained useful information for many hospitals that have yet to develop patient safety and infection control practices. As mentioned above, there are few hospitals performing these activities vigorously, and there is little information available about them in Japan. As such, this study opens the possibility for hospitals to learn from others with respect to patient safety and infection control practices. Finally, we also obtained potentially useful information for improving the reimbursement system and reallocating resources for the sustainability of quality healthcare delivery. Recently, the volume of patient safety and infection control activities remarkably increased, but the costs of these activities are not yet covered in the current payment system. Thus, the systematic and empirical findings presented here will be useful for the establishment of a safe and durable system of healthcare delivery.

Some limitations must be considered when interpreting the results of our study. First, hospitals selected in this study may not be representative of hospitals performing vigorous patient safety and infection control practices in Japan. In selecting participant hospitals, we utilized reputation, public relationship descriptions on their homepage, and magazine and newspaper articles. This was an attempt to ensure that participant hospitals would be among the most likely to have implemented the most rigorous practices. This was impossible to validate, however. Second, we could not get information on the activities performed by small groups, such as activity at the ward level. However, the aim of this study was to understand hospital-wide activities. Given time and financial limitations, the approach taken here was the most effective at achieving this objective.

Future studies are needed to further develop this framework in order to estimate the contribution of information technology systems, such as ordering systems and electronic medical charts. Since patient safety activities can be supplemented with information technology [16-18], it is necessary to study its contribution. Future studies are also needed to develop a framework to estimate activities surrounding informed consent, since this process is an important factor in informed decision making [19] and in reducing medical errors. Recently Japanese hospitals have begun to appropriate a greater number of medical professionals, and have allotted more time for the informed consent process. These changes require further examination.

## Conclusion

In this study, we developed a systematic framework to quantify hospital-wide activities for patient safety and infection control. We assessed the incremental volume of these activities in Japanese hospitals under the reimbursement containment policy. The government, as well as hospital decision makers that must understand resource allocation for patient safety and infection control activities, can benefit from this analytic framework and its empirical findings.

## Competing interests

The author(s) declare that they have no competing interests.

## Authors' contributions

YI conceived of the research. KH and HF collected the data and performed data analysis. KH wrote the first draft of the manuscript. All authors read, jointly revised and approved the final version of the manuscript.

## Pre-publication history

The pre-publication history for this paper can be accessed here:


